# miR-449 inhibits cell proliferation and is down-regulated in gastric cancer

**DOI:** 10.1186/1476-4598-10-29

**Published:** 2011-03-18

**Authors:** Tony Bou Kheir, Ewa Futoma-Kazmierczak, Anders Jacobsen, Anders Krogh, Linda Bardram, Christoffer Hother, Kirsten Grønbæk, Birgitte Federspiel, Anders H Lund, Lennart Friis-Hansen

**Affiliations:** 1 BRIC - Biotech Research & Innovation Centre and Centre for Epigenetics, University of Copenhagen, Copenhagen, Denmark; 2 Department of Clinical Biochemistry, Rigshospitalet, University of Copenhagen, Copenhagen, Denmark; 3 Bioinformatics Centre, Institute of Molecular Biology University of Copenhagen, Copenhagen, Denmark; 4 Department of Gastro-Intestinal, Rigshospitalet University of Copenhagen, Copenhagen, Denmark; 5 Department of Hematology, Rigshospitalet, University of Copenhagen, Copenhagen, Denmark; 6 Department of Pathology, Rigshospitalet, University of Copenhagen, Copenhagen, Denmark

## Abstract

**Background:**

Gastric cancer is the fourth most common cancer in the world and the second most prevalent cause of cancer related death. The development of gastric cancer is mainly associated with *H. Pylori *infection leading to a focus in pathology studies on bacterial and environmental factors, and to a lesser extent on the mechanistic development of the tumour. MicroRNAs are small non-coding RNA molecules involved in post-transcriptional gene regulation. They are found to regulate genes involved in diverse biological functions and alterations in microRNA expression have been linked to the pathogenesis of many malignancies. The current study is focused on identifying microRNAs involved in gastric carcinogenesis and to explore their mechanistic relevance by characterizing their targets.

**Results:**

Invitrogen NCode miRNA microarrays identified miR-449 to be decreased in 1-year-old *Gastrin *KO mice and in *H. Pylori *infected gastric tissues compared to tissues from wild type animals. Growth rate of gastric cell lines over-expressing miR-449 was inhibited by 60% compared to controls. FACS cell cycle analysis of miR-449 over-expressing cells showed a significant increase in the sub-G_1 _fraction indicative of apoptosis. ß-Gal assays indicated a senescent phenotype of gastric cell lines over-expressing miR-449. Affymetrix 133v2 arrays identified *GMNN*, *MET, CCNE2, SIRT1 *and *CDK6 *as miR-449 targets. Luciferase assays were used to confirm *GMNN*, *MET*, *CCNE2 *and *SIRT1 *as direct targets. We also show that miR-449 over-expression activated p53 and its downstream target p21 as well as the apoptosis markers cleaved CASP3 and PARP. Importantly, qPCR analyses showed a loss of miR-449 expression in human clinical gastric tumours compared to normal tissues.

**Conclusions:**

In this study, we document a diminished expression of miR-449 in *Gastrin *KO mice and further confirmed its loss in human gastric tumours. We investigated the function of miR-449 by identifying its direct targets. Furthermore we show that miR-449 induces senescence and apoptosis by activating the p53 pathway.

## Background

Gastric cancer is among the five most common cancers in the world and the second most prevalent cause of cancer-related deaths [1]. It is mainly, but not exclusively, caused by *H. Pylori *infection [2] as not all of *H. Pylori *infected persons develop tumours [3]. Other factors involved in the development of gastric cancer include the degree and type of the inflammatory response [2] as well as the levels of the *Gastrin *hormone [4,5]. Several studies have shown that both hypergastrinemia [6,7] and the lack of Gastrin [5] contribute to the pathogenesis of gastric cancer. Achlorhydria is a common feature of mouse models prone to developing metaplasia and cancer [6,8,9]. *Gastrin *knockout mice are achlorhydric [10], favouring a bacterial gastric overgrowth [11,12], and chronic bacterial gastric infections lead to gastric metaplasia which may progress into gastric cancer [6,12].

Since their discovery microRNAs, have been found implicated in a very wide range of normal and pathological processes [13]. MicroRNAs exert their regulatory functions posttranscriptionally by binding to partly complementary sequence motifs predominantly in the 3' UTR of target mRNAs resulting in mRNA destabilization and translational repression [14]. From a biological point of view, microRNAs are challenging objects to study as they regulate cohorts of target genes, which are not readily identified. From a therapeutical point of view, microRNAs are highly interesting as several studies have demonstrated the power of microRNAs as biomarkers and initial preclinical studies have established that microRNAs may be therapeutically targeted in vivo [15].

Profiling studies have evidenced microRNA deregulation in a broad spectrum of diseases including all major cancers [16]. MicroRNAs likely affect tumourigenic processes at two levels. Firstly, several studies have established pro-oncogenic or tumour-suppressive roles of individual microRNAs firmly linking these to cancer etiology as exemplified by miR-155, miR-10b and miR-21 [17-19]. Secondly, the microRNA regulatory system per se appears to have tumour suppressive functions as genetic ablation of key microRNA biogenesis factors, such as Dicer, strongly increase cancer susceptibility [20] and loss of function mutations have been identified in important microRNAs processing factors in human tumours [21-23].

In this study, we address the importance of microRNAs in gastric cancer taking advantage of the *Gastrin *knockout mouse model and *H. pylori *infection of wild type mice. We identify miR-449 as significantly down-regulated or lost in mouse models of gastric cancer as well as in primary human gastric tumours. Identification of mRNA targets reveals that this microRNA likely exerts tumour suppressive functions through the concerted regulation of a cohort of cancer-associated cell-cycle regulators including MET, GMNN, CCNE2, SIRT1, and HDAC1.

## Methods

### Mice

Three different age groups (12-16 weeks, 1 year or 1½ years) of wild type (wt) or *Gastrin *knockout (KO) mice were used. All mice were on a mixed 129/SvJ, C57BL/6J background, backcrossed at least four times to C57BL/6J [12]. The mice were kept under specific pathogen-free conditions and monitored according to the Federation of European Laboratory Animal Science Associations recommendation [24] with 12 h light, 12 h dark cycles.

### *H. pylori *infection

C57BL6/J mice (n = 10) were inoculated with a non-mouse-adapted clone of *H. Pylori *strain 67:21, originally isolated from an antral biopsy obtained from a Swedish female with gastric ulcer. The strain is VacA^+ ^and contains the entire Cag pathogenicity island (PAI) with genetic stability in the Cag PAI [25]. The mice were inoculated every second day (three times) during a 5-day period. DNA was extracted and analyzed for the presence of helicobacter species using a semi-nested polymerase chain reaction-denaturing gradient gel electrophoresis assay, specific for the genus helicobacter, as described previously [26]. A matched group of uninfected C57BL6/J mice were used as controls.

The stomachs of all mice were dissected into fundus and antrum prior to RNA extraction. All animal experiments were approved by the Danish Animal Welfare Committee (2005/562-40) and the Danish Forest and Nature Agency (20010077355/6).

### Mice antrum sections

Mice were sacrificed by cervical dislocation. The antrum was removed, washed gently in ice-cold PBS, frozen in liquid nitrogen and stored at -80°C until RNA extraction.

### Clinical samples analyses

Biopsies from gastric cancer and the adjacent normal tissues were obtained from patients undergoing surgery for gastric cancer at the Department of Gastrointestinal Surgery, Rigshospitalet. The inclusion took place in July to December 2008 and all patients provided signed, informed consent (Ethical committee approval H-B-2008-049) and Danish Data Protection Agency (2008-41-2138). The biopsies were placed in RNAlater (Ambion) in the operating room and subsequently frozen at -80°C until RNA extraction.

### RNA extraction and qPCR analyses

RNA was extracted using TRIzol (Invitrogen) according to manufacturer. miRNA expression profile was assessed using Taqman miRNA assays (Applied biosystems) for hsa/mmu-miR-449a and b, hsa/mmu-miR-34a, b and c and rnu44 or hsa/mmu-miR-191. Primer sequences for Affymetrix targets validation are listed in additional file 1, table S1.

### Cell culture

SNU638 and MKN74 were grown in RPMI-1640 (Gibco) with 10% FBS (Hyclone), 100U/ml penicillin and 100 μg/ml streptomycin (Invitrogen) and incubated at 37°C in 5% CO_2_. HCT116 cells (wt and p53-/- were grown in McCoy's 5A (Gibco) with 10% FBS (Hyclone), and 100U/ml penicillin and 100 μg/ml streptomycin (Invitrogen) and incubated at 37°C in 5% CO_2_. HEK293 and MEF cells (wt and p53-/-) were grown in DMEM (Gibco) with 10%FBS (Hyclone), 100U/ml penicillin and 100 μg/ml streptomycin (Invitrogen) and incubated at 37°C with 5% CO_2_.

### miRNA precursors and siRNA

miRNA precursors were purchased from Ambion, hsa-miR-449a (PM11521), hsa-miR-449b (PM11127) and hsa-miR-34a (PM11030).

### Cell growth analyses

SNU638 cells were seeded in 24-well plates and transfected the following day with 50nM miRNA duplex or siRNA using Lipofectamine 2000 (Invitrogen). Cells were fixed at indicated time points in 4% paraformaldehyde, stained in a 0.1% crystal violet solution, and resuspended in 10% acetic acid. Sample absorbance was measured at 620 nm.

### Cell cycle FACS analyses

SNU638 and MKN74 cells were seeded at 2 × 10^6 ^cells per 10cm plate and transfected with 50nM miRNA duplex (Ambion) using Lipofectamine 2000 (Invitrogen). Cells were harvested 48 and 72 hours post-transfection, stained for DNA content using propidium iodide (PI) and analyzed on a FACS Calibur flow cytometer (Becton-Dickinson). Briefly, cells were harvested by trypsinization and washed once with PBS before fixing over night in 70% EtOH. To stain the DNA, cells were pelleted, re-suspended in 100 μl EtOH and stained for 1 hour with 300 μl PI solution (0.05mg/ml PI, 20 μg/ml RNAse A in 0.1%BSA).

### Senescence analyses

SNU638 cells were seeded at 400.000 cells per 6-well-plate and transfected with 50nM miRNA duplex (Ambion) using Lipofectamine 2000 (Invitrogen). Four days post-transfection, cells were washed in PBS and fixed for 5 minutes at room temperature in 2% formaldehyde/0.2% glutaraldehyde. Cells were washed twice in PBS pH6.0 before being stained with fresh senescence associated β-Gal stain solution (1mg/ml 5-bromo-4-chloro-3-indolyl-βD-galactoside (X-Gal), 0.12mM K_3_Fe[CN]_6_, 0.12mM K_4_Fe[CN]_6_, 1mM MgCl_2 _in PBS pH6.0) overnight at 37°C without CO_2 _supply. Cells were washed once in PBS (pH6.0) and observed under the microscope.

### Antibodies and western blot analyses

SNU638 were seeded at 2 × 10^6 ^cells per 10 cm plate, transfected twice on two successive days with 50nM miRNA duplexes using Lipofectamine 2000 according to manufacturer (Invitrogen). Cells were harvested by trypsinization, washed once with PBS and lysed in RIPA buffer (150mM NaCl, 0.5% Sodium Deoxycholate, 0.1% SDS, 1% Igepal, 50mM Tris-HCl pH8, 2mM EDTA) supplemented with 1mM DTT, 1mM Pefabloc, 1mM NaV3, 10mM NaF and 1X complete mini protease inhibitor cocktail tablets. 25 μg of protein/lane were resolved on 4-20% NuPAGE Bis-Tris gels (Invitrogen) and transferred to a nitrocellulose membrane. Primary antibodies used were MET (Cell Signal 4560), MYC (Cell Signal 9402), GMNN (Santa Cruz Sc-53923), VCL (Sigma V9131), TP53 (Santa Cruz Sc-126), CDKN1A (Santa Cruz Sc-6246), CDK6 (Santa Cruz Sc-177), HDAC1 (Santa Cruz Sc-7872), CCNE2 (Cell Signal 4132), TUBB (Abcam ab11304), PARP (Cell Signal 9542) and Cleaved CASP3 (Cell Signal 9661).

### Microarray analyses

Small RNAs (< 200 nt) were isolated with Invitrogen PureLink miRNA Isolation Kit from fundic and antral tissue from 1) *Gastrin *KO mice and age and sex matched C57BL6/J control mice, and 2) C57BL6/J mice infected with *H. Pylori *and uninfected age and sex matched C57BL6/J control mice, (n = 4 for each group). The quality of isolated small RNAs was determined using the Small RNA Assay on an Agilent Bioanalyzer. 500ng of small RNA was labelled with Genisphere FlashTag Kit and hybridized to Invitrogen NCode Multi-Species miRNA Microarray V2 in a Maui hybridization station. Processed slides were scanned in an Agilent DNA microarray scanner. Resulting images was analyzed and ratio of median normalized using GenePix Pro 6.0. Four biological replicates were used for each comparison. Samples were hybridized to four arrays in a dual colour dye swap microarray experimental design. BRB ArrayTools were used for fold change and statistical calculations. Selected miRNA data from the array analysis were validated using TaqMan real-time PCR miRNA assays. Data will be deposited at ArrayExpress upon acceptance.

### mRNA arrays

SNU638 were transfected with 50 nM of miR-34a or miR-449b duplexes with siGLO siRNA used as negative control. Total RNA was extracted 24 hours post-transfection using TRIzol reagent. Affymetrix microarray analysis (HG-U133 Plus 2.0 human) was performed at the Microarray Center, Rigshospitalet, Copenhagen University Hospital. Experiments were run in either triplicates or quadruplicates. Data will be deposited at ArrayExpress upon acceptance.

### Vectors construction and reporter assays

The 3'UTRs of *HDAC1*, *SIRT1*, *MET*, *GMNN *and *CCNE2 *holding miR-449 binding sites were cloned downstream of the luciferase reporter in pMIR-REPORT vector system (Ambion). Quickchange site-directed mutagenesis kit (Stratagene) was used to induce two point mutations into the seed region. Mutagenesis primers sequences are listed in Additional file 1, table S1.

HEK293 cells were seeded in 96 well plates and transfected with 20nM miRNA precursor or scrambled siRNA control, 20-50ng of luciferase vector (pMIR-report) and 5ng of renilla vector (pRL-TK) using lipofectamine 2000 (Invitrogen). Cells were harvested 24 hours post transfection and luciferase activity was measured using Dual-Glo luciferase assay (Promega).

### Microarray analysis

Microarray expression data was processed using the 'affy' package in BioConductor [27]. Probe set intensities were summarized and quantile normalized using the BioConductor RMA and VSN packages. Differential expression was determined per probeset using a t-test. Probe sets were mapped to Ensembl transcripts (version 49) using mappings provided at BioMart. Probesets that mapped to two different Ensembl genes were discarded.

### Evaluating global down-regulation of microRNA target genes

The 3'UTRs, 5'UTRs and coding sequences of the transcripts were scanned for matching 6mer, 7mer and 8mer miRNA seed sites (complementary to position 2-7, 2-8, and 2-9 of the miRNA). Global analysis of miRNA target down-regulation was evaluated using the longest 3'UTR sequence per gene to avoid bias introduced by genes with many transcript isoforms. We discarded transcripts with 3'UTR sequences shorter than 50 nt. To globally evaluate if miRNA target genes were down-regulated after miRNA transfection, we tested the null hypothesis that the expression change distribution of miRNA targets (having a 7mer target site) was equal to the distribution of all expressed genes without predicted target sites using the non-parametric Wilcoxon rank-sum test. A similar approach was used to evaluate down-regulation of genes with miRNA target sites in coding regions and 5'UTRs of mRNAs.

### Exhaustive statistical assessment of words correlated with down-regulation

We used a previously published non-parametric rank-based statistic to exhaustively assess the correlation of word occurrences in 3'UTRs and the change in gene expression after miRNA transfection [28,29]. Genes were sorted by expression change induced by transfection of miR-34a or miR-449b, and the correlation with down-regulation was tested for all words of length 5-7 (N = 21 504).

### Statistical tests

Students t-test with Welch's correction.

## Results

### miR-449b is down regulated in the antrum of both *Gastrin *KO mice and *H. pylori *infected mice

**Figure 1:**
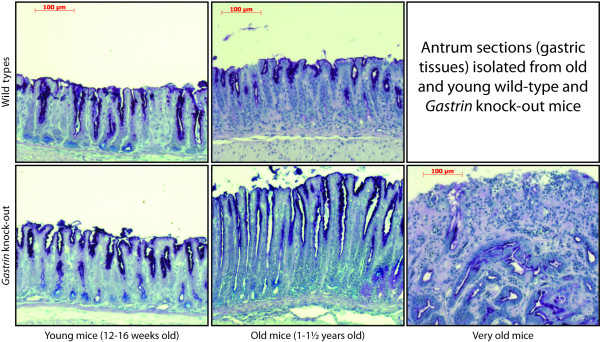
**Old *Gastrin *knockout mice develop gastric adenomas**. Antrum sections isolated from 12-16 weeks old mice (left panel), 12 to 18 months old mice (middle panel) and older than 18 months mice (right panel), from wild type (upper panel) and *Gastrin *knockout mice (lower panel). Sections show adenoma development in antrum tissues in the *Gastrin *knockouts compared to the wild types.

*Gastrin *knockout mice are achlorhydric with a tendency for developing antral hyperplasia and gastric adenomas over time (figure 1) [6,12]. In order to identify microRNAs deregulated during the development of gastric cancer, we examined miRNAs expression profiles in gastric neoplasias from *Gastrin *knockout mice using miRNA microarrays. As shown in table 1, 20 microRNAs were significantly deregulated in the knockout mice compared to wild type littermate controls, with three miRNAs differing more than two fold, miR-7 being up-regulated and miR-709 and miR-449b being down-regulated in the antrum of *Gastrin *knockout mice compared to wild types. To further confirm miR-449 deregulation during gastric cancer development, we examined its expression in wild type mouse antrum tissues infected with *H. Pylori*. Interestingly, miRNA arrays demonstrated a specific down-regulation of miR-449b in *H. Pylori *infected mice (table 2 and Additional file 1, figure S1).

**Table 1:** miRNAs deregulated in *Gastrin *knockout mice

miRNA Name	Log2-fold	p-value	miRNA Name	Log2-fold	p-value
mmu-miR-709	-1.73	7.0E-06	mmu-miR-422b	0.44	4.6E-02
mmu-miR-449b	-1.37	1.6E-03	mmu-miR-199a*	0.50	7.7E-03
mmu-miR-805	-1.01	7.5E-03	mmu-miR-25	0.51	1.6E-02
mmu-miR-706	-0.98	3.1E-03	mmu-miR-27b	0.56	5.1E-03
mmu-miR-467a	-0.88	2.8E-02	mmu-miR-182	0.56	1.8E-02
mmu-miR-696	-0.83	7.1E-03	mmu-miR-30a-3p	0.59	2.2E-02
mmu-miR-667	-0.66	2.3E-02	mmu-miR-10a	0.70	7.1E-03
mmu-miR-690	-0.28	1.7E-02	mmu-miR-152	0.70	2.3E-02
mmu-miR-18	0.29	3.7E-02	mmu-miR-1	0.77	7.1E-03
mmu-miR-143	0.34	2.3E-02	mmu-miR-7	1.06	4.9E-03

List of significantly deregulated miRNAs in gastric neoplasias from *Gastrin *Knockout mice compared to wild types.

**Table 2:** miRNAs deregulated in *H.Pylori *infected tissues

miRNA name	Fold change	p-value
mmu-miR-122a	-3.247	0.000788
mmu-miR-449b	-0.879	0.045214

List of significantly deregulated miRNAs in wild type mice antrum infected with *H. Pylori *compared to non-infected antrum.

### miR-449 inhibits cell cycle progression and induces senescence

**Figure 2:**
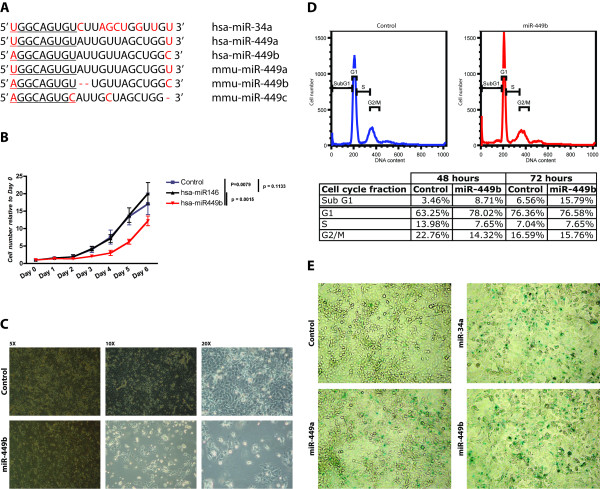
**miR-449 is part of the miR-34 family and inhibits cell proliferation**. **A **- miR-449 is part of the miR-34 family and is evolutionarily conserved. **B **- miR-449 re-introduction into human gastric cell lines (SNU638) inhibits cell proliferation (red line) compared to a scrambled control (blue line) and miR-146 control (black line). Error bars represent S.D. **C **- Visual inspection of cell proliferation inhibition and senescence-like phenotype upon miR-449 re-introduction into SNU638 cells (lower panel) compared to scrambled transfection control (upper panel). **D **- FACS cell cycle analysis showing sub-G_1 _accumulation of SNU638 cells 72 hours following miR-449 re-introduction (right histogram) compared to a scrambled transfection control (left histogram) The table shows cell accumulation in G_1 _fraction upon miR-449 re-introduction compared to scrambled RNA control at 48 hours post transfection, followed by a shift to the sub-G_1 _fraction at 72 hours post transfection indicative of cell death. **E **- Senescent phenotype of SNU638 cells upon miR-449 and miR-34a positive control re-introduction shown by acidic β-gal assay compared to RNA scrambled control.

Having demonstrated down-regulation of miR-449 expression in gastric cancers we wanted to examine the effect of re-expressing miR-449 in gastric cancer cell lines. Interestingly no noticeable expression of the miR-449 family was detected across a panel of gastric cell lines including SNU638, SNU5, SNU216, SNU601 and MKN74 sustaining the notion of miR-449 having tumour-suppressive functions (data not shown). The miR-449 family consists of miR-449a and b in humans and miR-449a, b and c in mice. Interestingly, they share the same seed sequence as the miR-34 family and are hence expected to regulate overlapping cohorts of target genes (figure 2a). To assess the function of miR-449 in gastric cell lines we re-introduced miR-449b in SNU638 and MKN74 cells. Compared to negative control microRNAs, re-introduction of miR-449b strongly affected the proliferation of SNU638 cells (figure 2b) and visual inspection of the cells indicated induction of apoptosis and cellular senescence (figure 2c, and Additional file 1, figure S2). Flow cytometric analysis of propidium iodide-stained cells transfected with miR-449b showed a G_1 _accumulation 48 hours after transfection, followed at 72 hours post transfection by an accumulation of cells in the sub G_1 _fraction suggestive of cell death (figure 2d). The induction of cellular senescence was confirmed by acidic beta gal staining using miR-34a as a positive control microRNA (figure 2e). To rule out cell line-specific effects, the functional consequences of miR-449 re-introduction in terms of cell cycle arrest were verified in MKN74 cells (Additional file 1, figure S3). Thus, re-introduction of miR-449 negatively affects proliferation of gastric cancer cell lines concomitant with the induction of senescence and apoptosis in concordance with miR-449 having tumour suppressive functions.

### The joint seed sequence of miR-449b and miR-34a induce highly correlated expression changes

To characterize the transcripts controlled by miR-449 and to see if miR-449 regulates different transcripts than miR-34a, SNU638 cells expression profiles were examined 24 hours post transfection of miR-449b or miR-34a and differentially expressed transcripts identified. We found that mRNAs with predicted miRNA target sites (7 mer seed site) in the 3'UTR were significantly down-regulated compared to mRNAs without predicted target sites after transfection of miR-449b (p < 1.2e-70, two-tailed Wilcoxon rank-sum test), (Additional file 1, figure S4a). mRNAs having predicted miRNA seed target sites in coding regions were also found to be significantly down-regulated (p < 9.9e-25), while mRNAs with sites in 5'UTRs were only marginally down-regulated (p < 5.3e-2). The expression changes induced by transfection of mature miR-449b and miR-34a were highly correlated despite divergence of the mature sequences outside the seed region (Pearson's correlation coefficient R = 0.94, p = 0), (Additional file 1, figure S4b). We exhaustively evaluated all oligonucleotides (words) of length 5-7 for correlation with down-regulation after miR-449b and miR-34a transfection (see methods). Consistent with many previous studies, this analysis revealed the shared miR-34a/449b seed site as the 3'UTR word most correlated with down-regulation in the two experiments (Additional file 1, figure S4c).

### miR-449 regulates numerous cell cycle controllers

**Figure 3:**
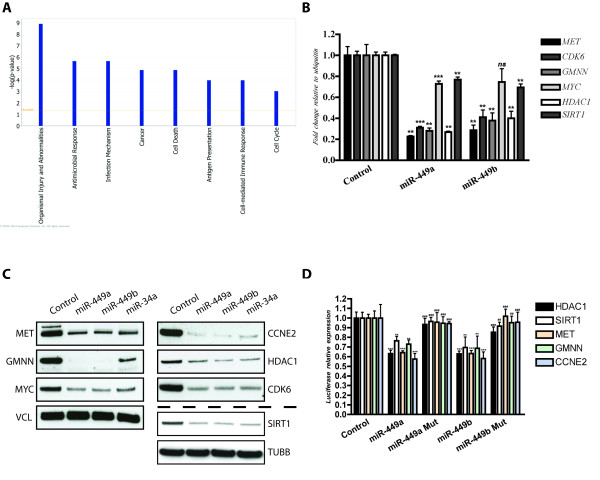
**miR-449 targets cell cycle controller genes**. **A **- Ingenuity Pathway Analysis (IPA) of deregulated genes upon miR-449 re-introduction into SNU638 cells showing enrichment for the gene categories cancer, cell death and cell cycle pathways among others. **B **- qPCR validation of Affymetrix arrays showing down-regulation of *MET*, *CDK6*, *GMNN*, *MYC *and *HDAC1 *upon miR-449 re-introduction compared to scrambled RNA controls. **C **- Western blot validation of down-regulated genes upon miR-449 re-introduction into SNU638 cells compared to scrambled RNA controls. Vinculin (VCL) and tubulin beta (TUBB) were used as loading controls **D **- verification of direct and functional target binding using luciferase constructs holding wild type 3'UTRs and mutated 3'UTRs (two mutations in miR-449 binding site), * indicates statistical significance in luciferase expression between wild type 3'UTRs transfected with miR449a/b compared to RNA scrambled control, # indicates statistical difference in luciferase expression between wild type 3'UTRs compared to mutant 3'UTRs transfected with miR-449a and miR-449b. "ns" not significant p value > 0.05, "*" or "#" significant 0.01 < p value < 0.05, "**" or "##" very significant 0.01 < p value < 0.001, "***" or "###" extremely significant p value < 0.001

The expression profiles were used to identify differentially expressed transcripts in cells transfected with miR-449b or controls (Additional file 1, table S2). A pathway activation analysis based on the differentially regulated transcripts demonstrates that miR-449 mainly controls transcripts coding for proteins involved in cell damage responses, cell cycle control, inflammation and cancer pathways (figure 3a). Focusing on a set of putative target genes with well-established roles in tumourigenesis, we confirmed down-regulation by miR-449 of met proto oncogene (*MET*), cyclin dependent kinase 6 *(CDK6)*, geminin *(GMNN)*, myelocytomatosis viral oncogenes homolog *(MYC)*, sirtuin 1 (*SIRT1*) and histone deacetylase 1 *(HDAC1) *at the transcript level (figure 3b). Western blot analyses confirmed the ability of miR-449 to down-regulate MET, GMNN, MYC, SIRT1, cyclin E2 (CCNE2) and HDAC1 at the protein level to an extent similar to that achieved by re-introduction of miR-34a (figure 3c). For a subset of target genes including *MET*, *GMNN, CCNE2*, *SIRT1 *and *HDAC1*, we confirmed direct interaction of miR-449 with the target gene 3' UTR using luciferase assay (figure 3d).

Hence, miR-449 directly targets cell cycle regulator genes consistent with a tumour suppressor function and with the cell cycle arrest observed upon miR-449 re-introduction into cancer cell lines.

### miR-449 induces p53 expression but is not regulated by p53

**Figure 4:**
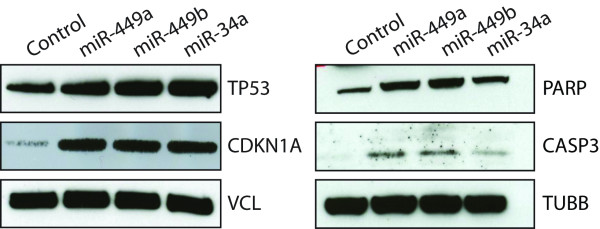
**miR-449 activates the p53 pathway**. Western blot analysis showing an increase of the p53 protein upon miR-449 and positive control miR-34a re-introduction into SNU638 cells compared to RNA scrambled control as well as an activation of the p53 downstream target p21 and apoptosis markers cleaved CASP3 and PARP. Vinculin (VCL) and tubulin beta (TUBB) were used as loading controls.

As miR-34a was previously found to function downstream of p53 [30-33], we analyzed if also miR-449a/b were linked to p53. This was furthermore spurred by the presence of a putative p53 binding site 10 kb upstream from human miR-449 (data not shown). We therefore induced p53 by DNA damage using UV or 5-fluorouracil (5FU) in four different systems, HCT116 and MEF wild type and p53 knockout cells (Additional file 1, figure S5a). However, no significant change in miR-449 expression was detected after p53 pathway activation (Additional file 1, figure S5b). In conclusion, we found no evidence that miR-449 is a transcriptional target of p53. On the other hand, we found that miR-449a/b is able to induce activation of p53, activation of p53 response genes such as p21 and the induction of apoptosis as evidenced by cleavage of caspase 3 (CASP3) and poly (ADP-ribose) polymerase 1 (PARP) (figure 4). Towards understanding the mechanism by which miR-449 does this we examined the effect of miR-449 on SIRT1 and HDAC1. SIRT1 and HDAC1 are deacetylases which inhibit, among others, the activation of p53 and miR-34 has been shown to repress SIRT1[34]. We validated the specific binding of miR-449 to SIRT1 and HDAC1 using 3'UTR luciferase assays (figure 3d).

Hence, we speculate that miR-449 induces apoptosis by inhibiting the histone deacetylase HDAC1 and SIRT1 leading to the p53 pathway activation thus the induction of apoptosis markers cleaved CASP3 and PARP.

### miR-449 is down-regulated in human gastric cancers

**Figure 5:**
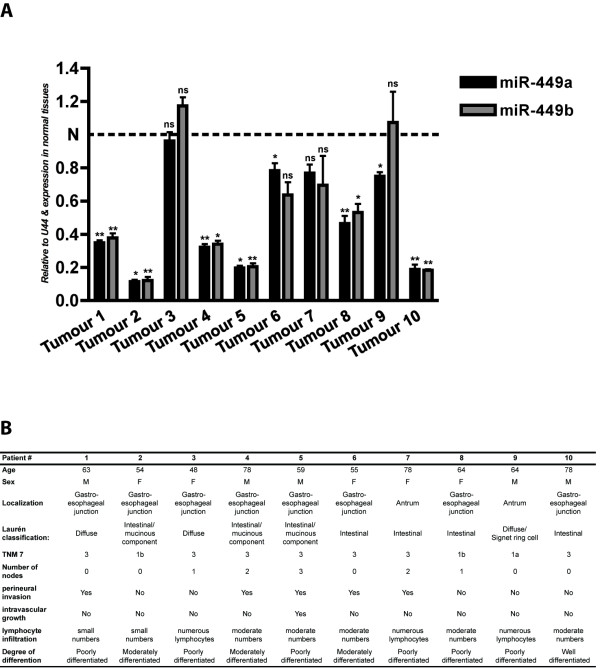
**miR-449 is down regulated in human gastric cancers**. qPCR analysis (upper panel) showing down-regulation of miR-449 expression in 8 gastric cancer tissues compared to miR-449 expression in sample-matched controls (dotted line). U44 was used as endogenous control. Table showing clinical information of patients (lower panel). "ns" not significant p value > 0.05, "*" significant 0.01 < p value < 0.05, "**" very significant 0.01 < p value < 0.001, "***" extremely significant p value < 0.001

To evaluate the importance of miR-449 in human malignancies we next examined the expression of miR-449 in 10 gastric cancer biopsies. Importantly, we found both miR-449a and b to be significantly down-regulated or absent in 8 out of 10 primary gastric cancers. Furthermore, the expression of miR-449a and b seem to be co-regulated (figure 5a). We did not find any correlation between the reduction in miR-449 expression and clinical characteristics of the cancer (figure 5b). Analyses of the genomic DNA from the tumours found no evidence for loss or hyper-methylation of the miR-449 loci using methylation-specific melting curve analysis (MS-MCA) indicating transcriptional down-regulation of expression (data not shown). Hence, in agreement with data from two mouse models of gastric inflammation and hyperplasia, the expression of miR-449 is down-regulated in human gastric cancers.

## Discussion

Gastric cancer is a highly lethal malignancy with more than 21,500 new cases each year in the United States alone [35]. The disease is often detected late and the 5-year survival rate is consequently below 20% [36]. It is therefore important to understand the etiology and progression stages of the disease. The importance of the bacterial, environmental and host genetic risk factors in gastric carcinogenesis have been studied, however less is known about the molecular progression of the disease [37,38]. Among others, p53 pathway inactivation is reported in 30-60% of gastric cancers [39,40] and recent studies suggest *H. pylori *direct modulation of the p53 gene or its downstream targets [41]. Another common alteration in gastric cancer is the perturbation of the cell cycle control via the over expression of Cyclin E1, which is associated with tumour aggressiveness and lymph node metastasis [42,43]. In contrast to our current understanding of the role of tumour suppressors and cell cycle factors, the knowledge about the post transcriptional changes affecting gene expression in gastric cancer is still incomplete.

Using the *Gastrin *knockout mouse model for gastric cancer [6,12], we identify 20 deregulated microRNAs of which 3 were more than 2-fold deregulated relative to their levels in normal gastric mucosa. As infection with *H. pylori *has been causally linked to gastric cancer [44,45], we next examined changes in microRNA expression levels following infection of wild type mice with *H. Pylori*. Interestingly, miR-449b was the only miRNA significantly deregulated in both mouse models. To establish a causal relationship between miR-449 deregulation and cancer-relevant parameters, such as cell cycle regulation, apoptosis and senescence, we over-expressed miR-449 in gastric cancer cell lines and observed a significant down-regulation of proliferation coupled with up-regulation of the acidic beta-gal senescence marker and induction of apoptosis. Importantly, analyses of primary gastric tumours from patients clearly documented a tumour-specific down-regulation of miR-449 also in humans. Previously, a number of studies have demonstrated deregulation of miRNA in gastric cancer [46-50] and the importance of the entire microRNA system has been documented in both gastric and colon cancer by the presence of cancer-specific mutations in the key RISC components *AGO2 *and *TNRC6A *[51]. Furthermore, microRNAs are likely of important prognostic value in gastric cancer [52-54]. The present study represents the first report demonstrating cancer-related down-regulation of miR-449 in both mouse models for gastric cancer and in primary human gastric tumours. Beside gastric cancer, the expression of miR-449 has also been found to be reduced in several cell lines [55] and in prostate cancer, where it was found to target *HDAC1 *and induce growth arrest following over-expression in prostate cancer cells [56]. In contrast, the expression of miR-449 has been reported to be increased in endometrioid adenocarcinoma [57] and melanoma in young adult patients [58]. The expression of miR-449 was also increased in skeletal muscle damage and regeneration [59].

To unveil molecular links between the loss of miR-449 and cancer progression or initiation we experimentally identified a number of direct mRNA targets using transcriptional profiling and extensive bioinformatics analysis. A series of the putative miR-449 targets were subsequently validated at endogenous level using western blotting and quantitative PCR and their direct regulation by miR-449 was established using heterologous reporter constructs and binding site-specific mutation studies. Prominent validated targets include *CDK6*, *MYC*, *CCNE2*, *MET *and *GMNN *and we furthermore validated the regulation of *HDAC1 *and *SIRT1 *also in gastric cancer cells. Hence, the cancer-specific loss or down-regulation of miR-449 in gastric cancer can likely be explained by the connection to key cell cycle regulators. During the search for miR-449 targets we also identified several growth factors (*AREG*, and *KITLG*) and growth factor receptors, such as *MET*, as targets. This suggests that deregulation of miR-449 not only leads to deregulated control of cell cycle proteins but also of growth factors and their receptors. Thus, another important property of miR-449 could be as a regulator of signals important for growth and migration/invasion.

Interestingly, miR-449 was recently shown to operate under the control of E2F1 [55,60]. This is highly interesting as it places miR-449 at a key node in a feed-back loop in which E2F1 activates the transcription of miR-449 that in turn targets *CDC25A *and *CDK6*. Reduced levels of CDC25A and CDK6 decreases the phosphorylation of pRB and subsequently inhibits the release of E2F1 [55,60,61]. Hence, aside from the pro-oncogenic effects of up-regulation of MYC, MET, CCNE2 and other direct targets, loss of miR-449 may result in increased E2F1 activity.

Many of the direct mRNA targets for miR-449 identified in this study are also targets of miR-34a and miR-449 and miR-34a belong to the same family of miRNAs as they share the same seed sequence. In accordance, miR-34a has been demonstrated to act as an important tumour-suppressor miRNA and reduced expression of miR-34a has been reported for neuroblastoma [62] and many other cancers [63]. In addition, the miR-34a gene is activated by p53 following DNA damage [30-33] and by ELK1 following over-expression of activated B-RAF [64].

In the present study, transcriptional profiling demonstrated that over-expression of miR-449 or miR-34a results in identical transcriptome changes. While this is underlining the dominant importance of the seed sequence in this type of over-expression study it also points to a limitation in the experimental approach. While miR-449 was clearly down-regulated or lost in the analyzed mouse tumour samples no clear tendency for loss or down-regulation of miR-34a was observed (data not shown). This indicates that these microRNAs may have more deviant functions *in vivo *than suggested by the over-expression studies.

Finally, we examined the relationship between the p53 tumour suppressor and miR-449. As miR-34a has been firmly placed downstream from p53 [30-33] it was relevant to test if the same was the case for miR-449. In agreement with other studies [55,60], we did not observe a p53-dependent regulation of miR-449 in gastric cancer cells as well as in primary human and mouse fibroblasts. However, in agreement with previous findings for miR-34a, we find that miR-449 regulates the expression of p53 [31,32] as over-expression of miR-449 resulted in a potent up-regulation of p53 subsequently resulting in activation of p21 and induction of apoptosis markers, such as cleaved CASP3 and PARP as previously reported [60].

In summary, we have found that miR-449 may act as a tumour suppressor and is lost in gastric cancer. Its re-introduction into cancer cell lines leads to inhibition of cell proliferation by targeting different cell cycle regulators. We also found that re-introduction of miR-449 induces senescence and apoptosis. Hence, this study further underlines the importance of miRNAs in cancer and points to an important function for miR-449 in gastric cancer.

## Competing interests

The authors declare that they have no competing interests.

## Authors' contributions

TB performed cell cycle and senescence studies, targets validation and direct targets detection studies, p53 activation studies and miR-449 expression studies, conducted data analyses, contributed in designing the study and in writing the manuscript. EF performed the miRNA array, target identification experiments, growth assays and direct targets detection studies. miR-449 expression studies, conducted data analyses and contributed in writing the manuscript. AJ performed the bioinformatics on target identification, contributed in writing the manuscript. AK supervised the bioinformatics on target identification, contributed in writing the manuscript. LB collected the normal and tumour patient samples. CH performed the bio-informatical analysis of the miRNA arrays. KG performed the methylation assays. BF performed the pathologicalscoring of the pathological samples. AHL participated in designing the study and in writing the manuscript. LFH participated in designing the study and in writing the manuscript. All authors have read and approved the final manuscript.

## Supplementary Material

Additional file 1 **Figure S1 - miR-449 is down-regulated in *Gastrin *knock out mice compared to wild type**. qPCR analysis of miR-449 expression in *Gastrin *knock out gastric tissues compared to relative expression in wild type gastric tissues, miR-449 is significantly down-regulated (p = 0.04) in *Gastrin *knock out tissues compared to wild types. **Figure S2 - miR-449 inhibits cell proliferation in human gastric cancer cell line MKN74**. Visual inspection of human gastric cancer cell line (MKN74) upon miR-449 re-introduction (lower panel) showing a decrease in cell proliferation as well as a senescent like phenotype compared to scrambled RNA control (upper panel) **Figure S3 - miR-449 induces cell death in human gastric cancer cell line MKN74**. FACS cell cycle analysis of MKN74 cell line upon miR-449 re-introduction (right histogram) showing an increase in the sub-G_1 _fraction indicative of cell death compared to scrambled RNA control (left histogram), table showing percentage of cell accumulation in G_1 _fraction 48 hours post miR-449 re-introduction followed by cell shift to the sub-G_1 _fraction 72 hours post transfection compared to RNA scrambled control. **Figure S4 - miR-449b and miR-34a induce highly correlated expression changes**. **A **- Chart showing significant down-regulation (p < 1.2e-70) of mRNAs with predicted miR-449 seed match in their 3'UTR (red line) compared to mRNAs lacking the seed match (black line). **B **- Chart showing high correlation of expression changes upon re-introduction of miR-449b or miR-34a into SNU638 cells with a Pearson's correlation coefficient of r = 0.94, p = 0. **C **- Word analysis showing shared miR-449b/34a seed site correlating with gene down-regulation. **Figure S5 - miR-449 expression is p53 independent**. **A **-Western blot showing p53 induction in HCT116 wild types and p53 knockouts using 5FU. **B **- qPCR analyses of miR-449a and miR-449b post p53 induction. No significant change is observed. **Table S1 - primer sequences**. **Table S2 - list of genes deregulated upon miR-449 re-introduction**. Affymetrix top down-regulated genes upon miR-449 re-introduction into SNU638 cells. Click here for file
