# Supplementary material for: miR-449 inhibits cell proliferation and is down-regulated in gastric cancer
Source: Mol Cancer. 2011 Mar 18;10:29. doi: 10.1186/1476-4598-10-29 (PMC3070685; doi:10.1186/1476-4598-10-29)

**Additional file 1**

Figure S1


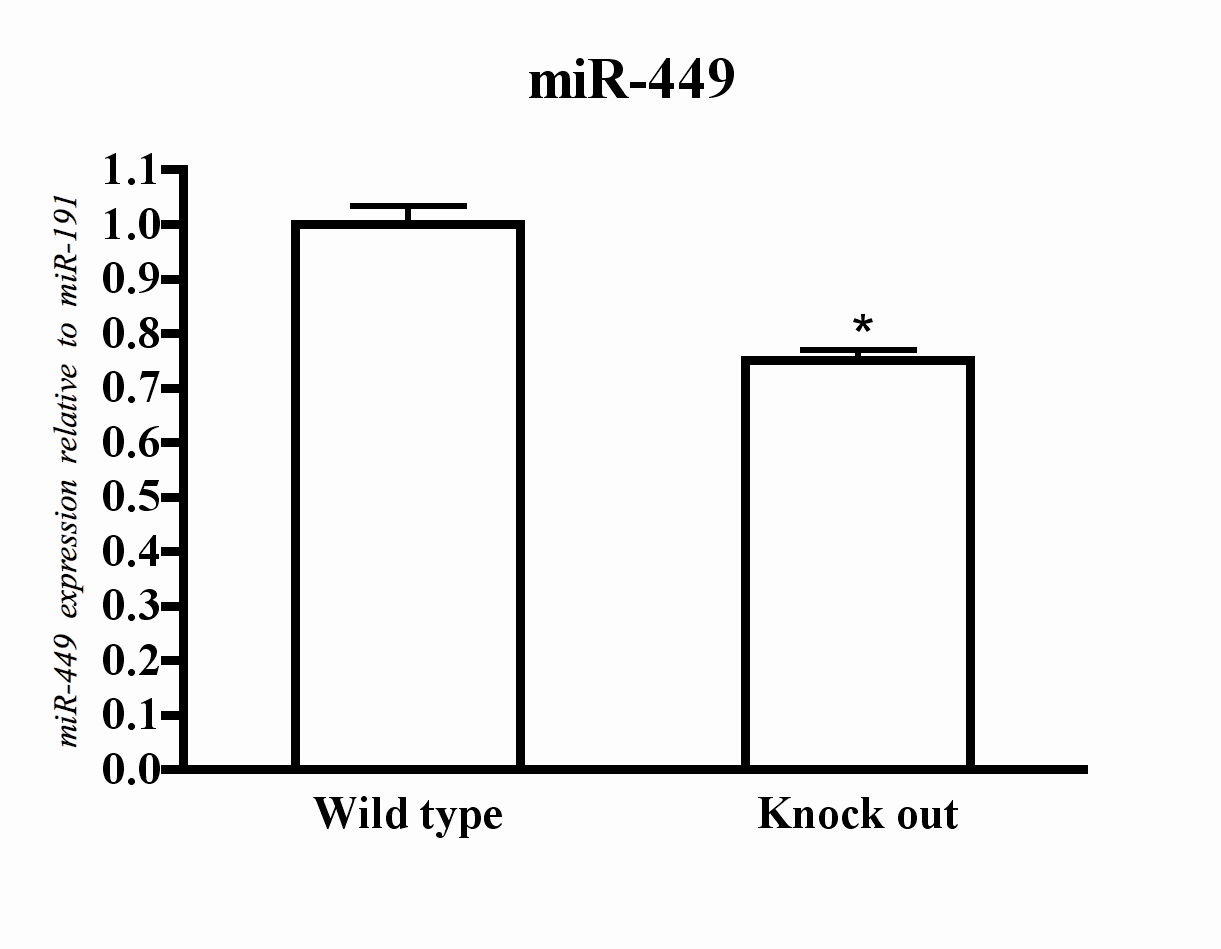


Figure S2


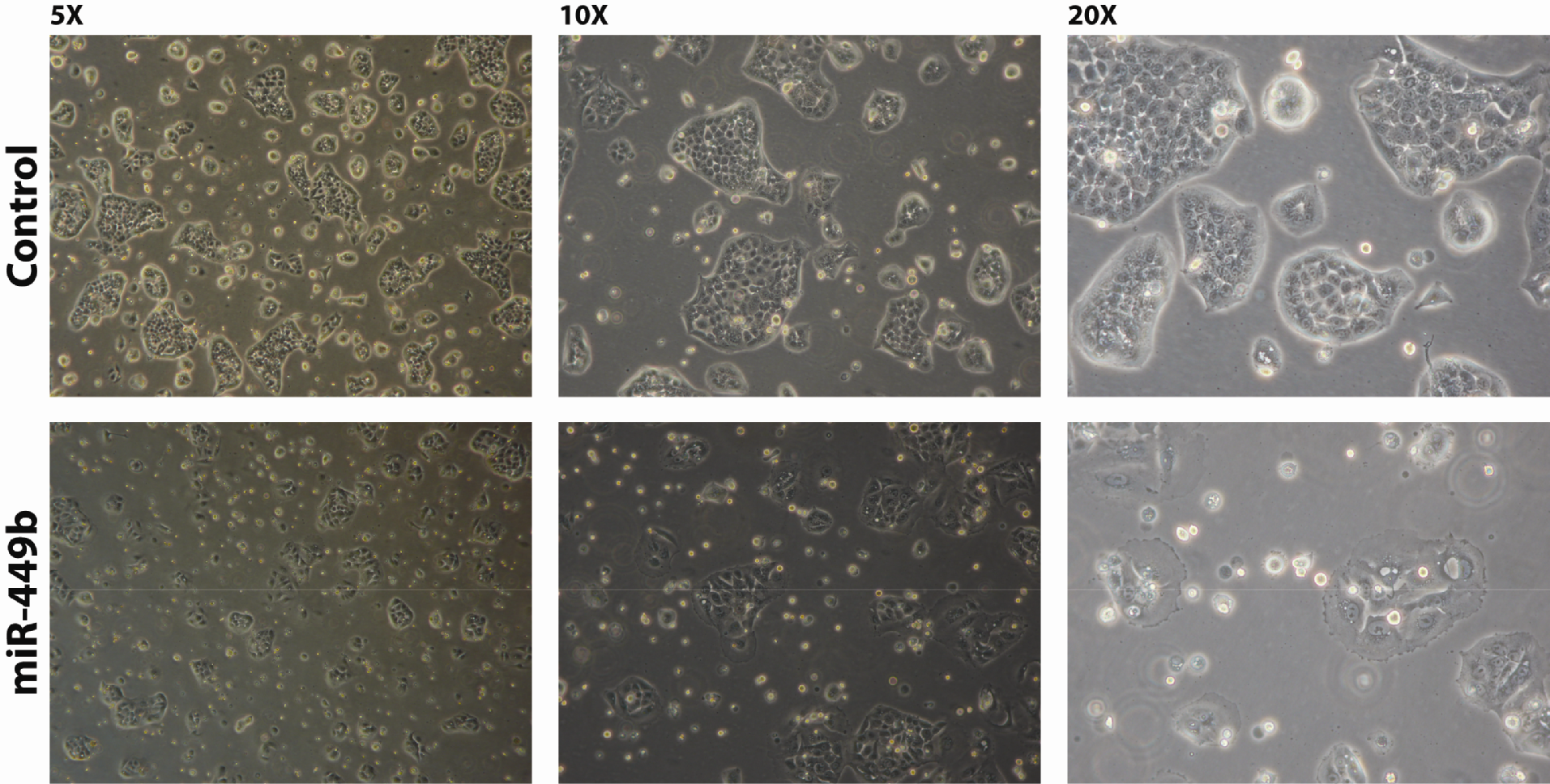


Figure S3


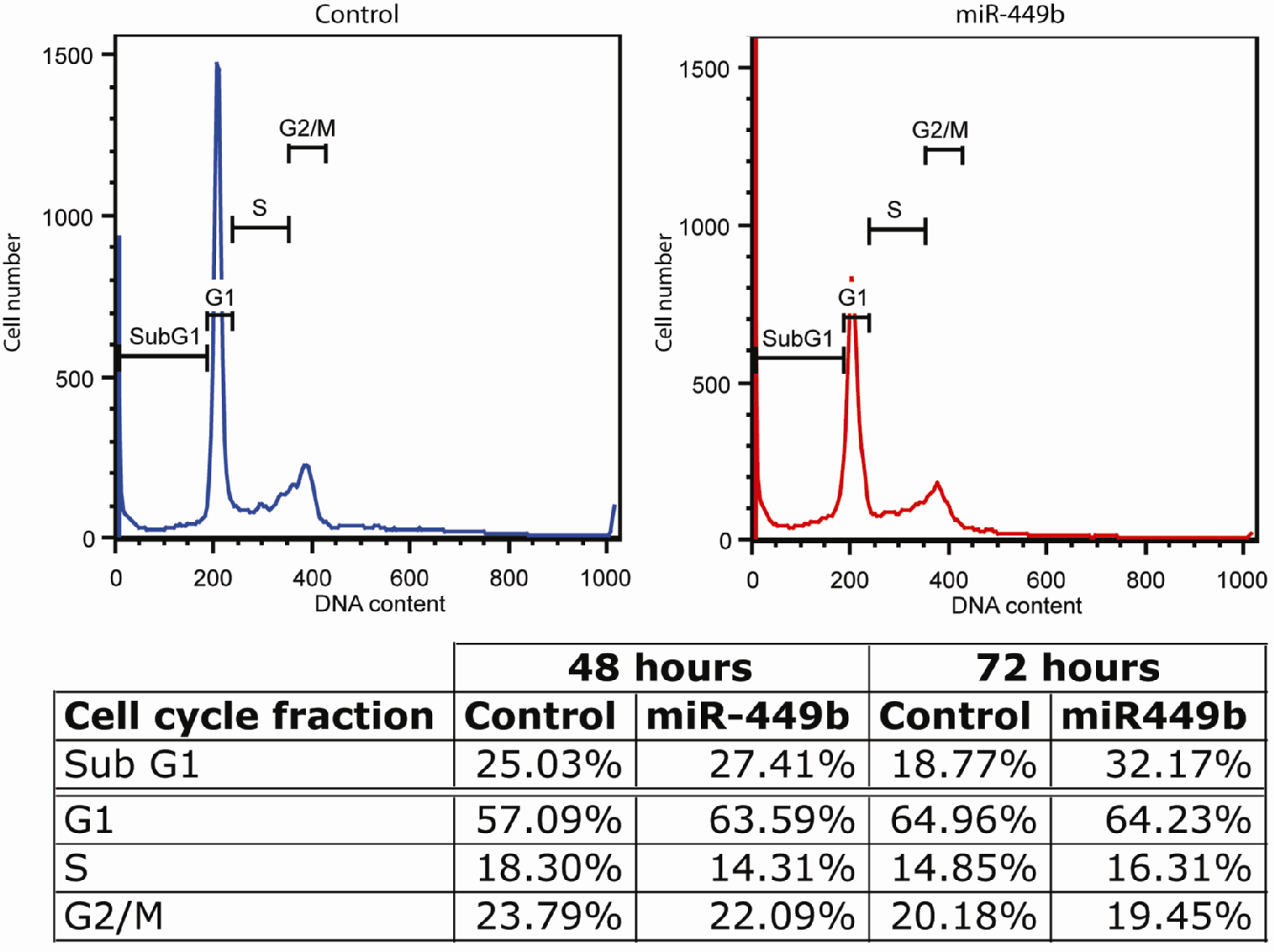


Figure S4


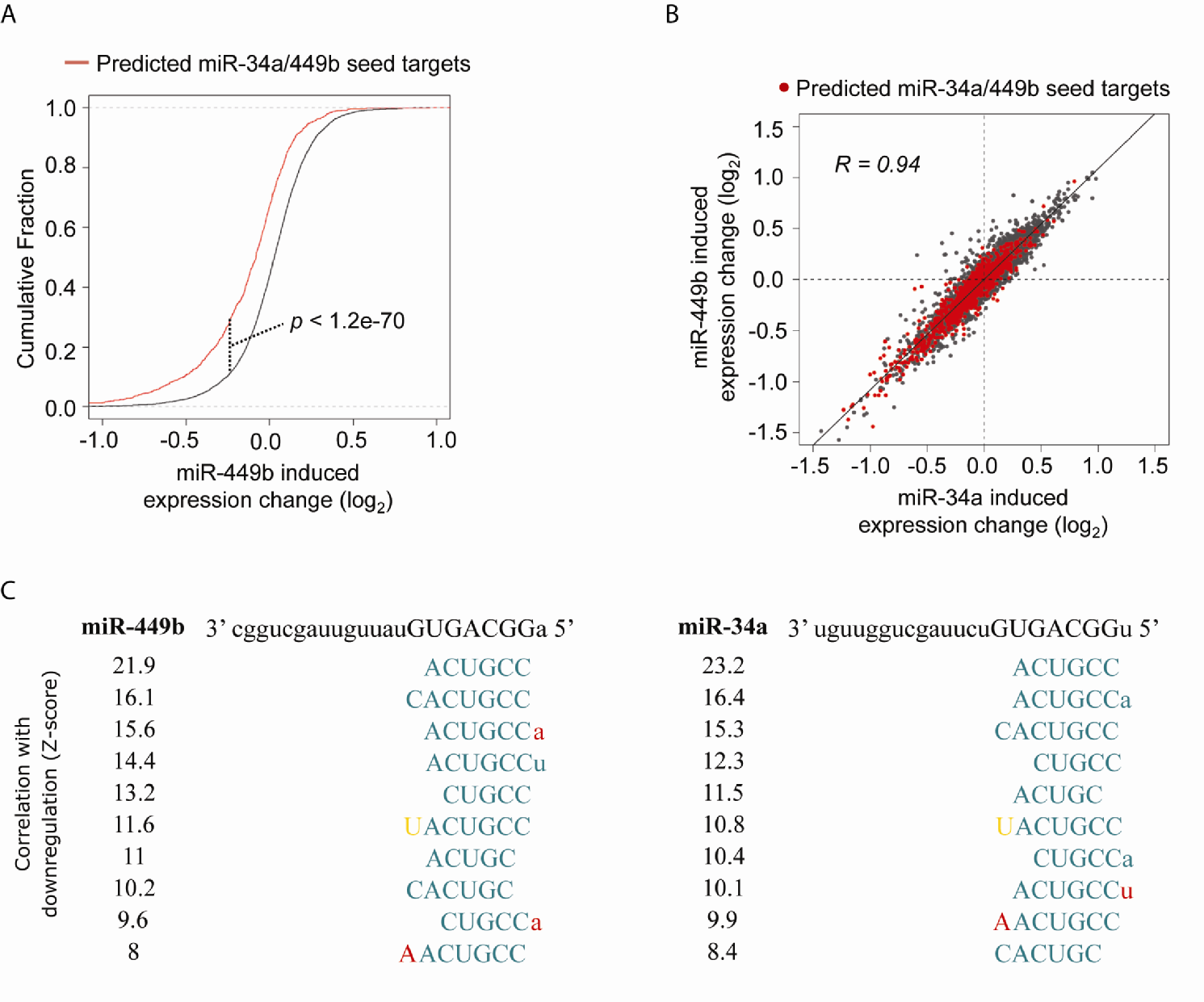


Figure S5


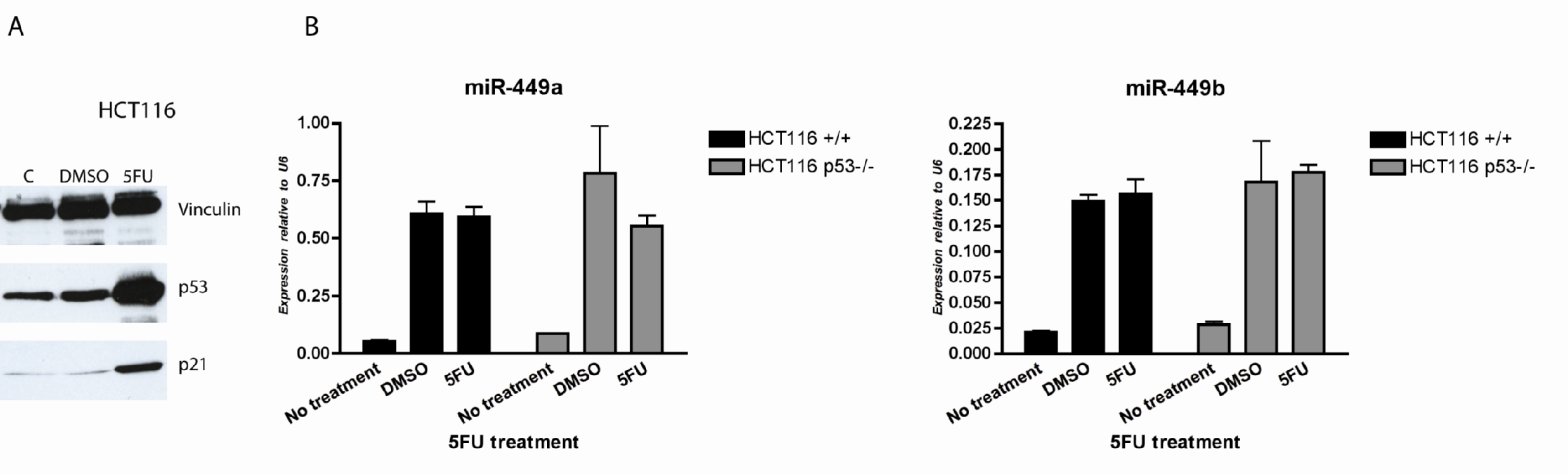


Table S1

Table S1 – primer sequences

| **Primers for Affymetrix array validation** | | |
| --- | --- | --- |
| Primer | Fw sequence | Rev sequence |
| *MET* | GCAGGTTGTGGTTTCTCGAT | TGCAGGACTGGAAATGTCTG |
| *CDK6* | GCCTATGGGAAGGTGTTCAA | TGACACTGTGCACACATCAAA |
| *GMNN* | CGCGTCCTACTTTGACAGC | GCGGACAGCTCATTTTCTCT |
| *MYC* | GAGGCTATTCTGCCCATTTG | CACCGAGTCGTAGTCGAGGT |
| *HDAC1* | CGAATCCGCATGACTCATAA | TCTCTGCATCTGCTTGCTGT |
| **Mutagenesis primers** | | |
| *MET* | TGATTCTTCTAAGAATTAGATACTTGTGACTCCCTATACCTGCAGCTG | CAGCTGCAGGTATAGGGAGTCACAAGTATCTAATTCTTAGAAGAATCA |
| *CCNE2* | TAGCCAATTCACAAGTTAGACTCCCATTCTGATTTTAAAACTT | AAGTTTTAAAATCAGAATGGGAGTCTAACTTGTGAATTGGCTA |
| *HDAC1* | CCTCAAGTGAGCCAAGAAAGACTCCCTGCCCTC | AGAGGGCAGGGAGTCTTTCTTGGCTCACTTGAGG |
| *SIRT1* | CACCCAGCTAGGACCATTAGACCCAGAGAAAAAAATCGTA | TACGATTTTTTTCTCTGGGTCTAATGGTCCTAGCTGGGTG |
| *GMNN* | TTTGACTGTTGAGAATTTTAGTCCCGAAGTTTACCTCCACTAGT | ACTAGTGGAGGTAAACTTCGGGACTAAAATTCTCAACAGTCAAA |

Table S2


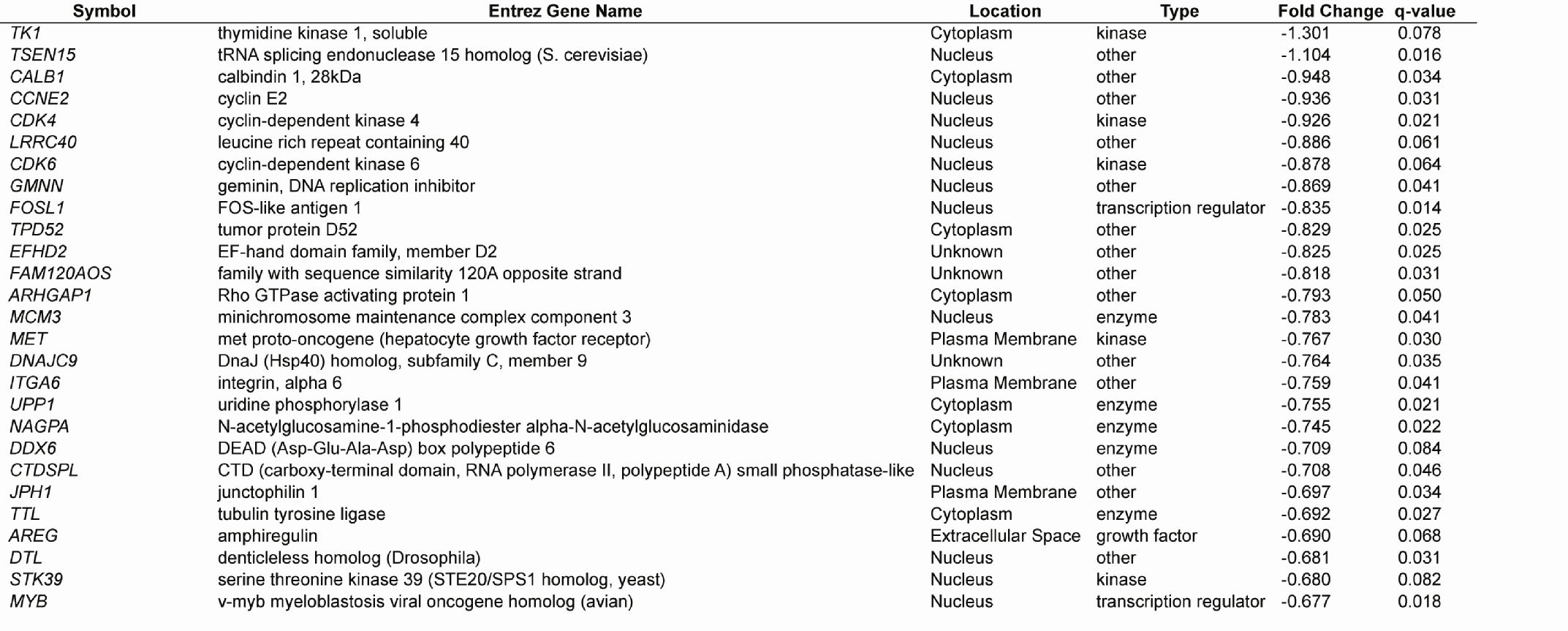

Supplement: Additional file 1 — Figure S1 - miR-449 is down-regulated in Gastrin knock out mice compared to wild type. qPCR analysis of miR-449 expression in Gastrin knock out gastric tissues compared to relative expression in wild type gastric tissues, miR-449 is significantly down-regulated (p = 0.04) in Gastrin knock out tissues compared to wild types. Figure S2 - miR-449 inhibits cell proliferation in human gastric cancer cell line MKN74. Visual inspection of human gastric cancer cell line (MKN74) upon miR-449 re-introduction (lower panel) showing a decrease in cell proliferation as well as a senescent like phenotype compared to scrambled RNA control (upper panel) Figure S3 - miR-449 induces cell death in human gastric cancer cell line MKN74. FACS cell cycle analysis of MKN74 cell line upon miR-449 re-introduction (right histogram) showing an increase in the sub-G1 fraction indicative of cell death compared to scrambled RNA control (left histogram), table showing percentage of cell accumulation in G1 fraction 48 hours post miR-449 re-introduction followed by cell shift to the sub-G1 fraction 72 hours post transfection compared to RNA scrambled control. Figure S4 - miR-449b and miR-34a induce highly correlated expression changes. A - Chart showing significant down-regulation (p < 1.2e-70) of mRNAs with predicted miR-449 seed match in their 3'UTR (red line) compared to mRNAs lacking the seed match (black line). B - Chart showing high correlation of expression changes upon re-introduction of miR-449b or miR-34a into SNU638 cells with a Pearson's correlation coefficient of r = 0.94, p = 0. C - Word analysis showing shared miR-449b/34a seed site correlating with gene down-regulation. Figure S5 - miR-449 expression is p53 independent. A -Western blot showing p53 induction in HCT116 wild types and p53 knockouts using 5FU. B - qPCR analyses of miR-449a and miR-449b post p53 induction. No significant change is observed. Table S1 - primer sequences. Table S2 - list of genes deregulated upon miR-449 re-i [file 1476-4598-10-29-S1.DOC]
